# Intermittent Hypoxia Up-Regulates Gene Expressions of *Peptide YY (PYY), Glucagon-like Peptide-1 (GLP-1), and Neurotensin (NTS)* in Enteroendocrine Cells

**DOI:** 10.3390/ijms20081849

**Published:** 2019-04-15

**Authors:** Ryogo Shobatake, Asako Itaya-Hironaka, Akiyo Yamauchi, Mai Makino, Sumiyo Sakuramoto-Tsuchida, Tomoko Uchiyama, Hiroyo Ota, Nobuyuki Takahashi, Satoshi Ueno, Kazuma Sugie, Shin Takasawa

**Affiliations:** 1 Department of Neurology, Nara Medical University, 840 Shijo-cho, Kashihara, Nara 634-8522, Japan; rshobatake@naramed-u.ac.jp (R.S.); sueno@naramed-u.ac.jp (S.U.); ksugie@naramed-u.ac.jp (K.S.); 2 Department of Neurology, Nara City Hospital, 1-50-1 Higashikidera-cho, Nara, Nara 630-8305, Japan; takahashin@nara-jadecom.jp; 3 Department of Biochemistry, Nara Medical University, 840 Shijo-cho, Kashihara, Nara 634-8521, Japan; iasako@naramed-u.ac.jp (A.I.-H.); yamauchi@naramed-u.ac.jp (A.Y.); m.makino@naramed-u.ac.jp (M.M.); ssumiyo@naramed-u.ac.jp (S.S.-T.); uchiyama0403@naramed-u.ac.jp (T.U.); hiroyon@naramed-u.ac.jp (H.O.)

**Keywords:** sleep apnea syndrome, intermittent hypoxia, enteroendocrine cells, peptide YY, glucagon-like peptide-1, neurotensin, appetite

## Abstract

The patients with sleep apnea syndrome are exposed to intermittent hypoxia (IH) during sleep. We previously demonstrated the IH-induced up-regulation of the mRNA levels of anorexigenic peptides proopiomelanocortin (POMC), and cocaine- and amphetamine-regulated transcript (CART) in human neuronal cells. Appetite is regulated not only by the central nervous system but also by the peptides from gastrointestinal tract. Here, we investigated the effects of IH on the gene expression(s) of appetite-inhibiting gut hormones. Human enteroendocrine Caco-2 and mouse STC-1 cells were exposed to IH [64 cycles of 5 min hypoxia (1% O_2_) and 10 min normoxia (21% O_2_)] or normoxia for 24 h. Real-time RT-PCR revealed that IH significantly increased the mRNA levels of *peptide YY* (*PYY*), *glucagon-like peptide-1* (*GLP-1*), and *neurotensin* (*NTS*) in Caco-2 and STC-1 cells. ELISA showed that the concentrations of PYY, GLP-1, and NTS in the culture medium were significantly increased by IH. The mRNA levels of *PYY*, *GLP-1*, and *NTS* were significantly up-regulated even in normoxia by Trichostatin A (TSA) and were significantly decreased even in IH by 5-azacytidine (5AZC), suggesting that IH increases *PYY*, *GLP-1*, and *NTS* mRNAs via alterations in the chromatin structure in enteroendocrine cells. IH might have an anorexigenic influence on the enteric nervous system.

## 1. Introduction

Sleep apnea syndrome (SAS) is a common disorder characterized by repetitive episodes of oxygen desaturation during sleep, the development of daytime sleepiness, and the deterioration of quality of life [1]. During sleep, the patients with SAS undergo serious recurrent apnea, being exposed to alternating low oxygen pressure and normal oxygen pressure, that is, intermittent hypoxia (IH) [2]. Accumulating evidence indicates a strong relationship between obesity and SAS [3]. We previously investigated the effect of IH on the regulation of appetite in SAS patients by analyzing the expression(s) of major appetite regulatory neuropeptide and receptor genes such as *proopiomelanocortin* (*POMC*), *cocaine- and amphetamine-regulated transcript* (*CART*), *galanin* (*GAL*)*, galanin-like peptide* (*GALP*), *ghrelin* (*GHRL*), *pyroglutamylated RFamide peptide* (*QRFP*), *agouti-related peptide* (*AGRP*), *neuropeptide Y* (*NPY*), and *melanocortin 4 receptor* (*MC4R*) in IH-exposed human neuronal cells and demonstrated that IH up-regulated the mRNA levels of anorexigenic peptides POMC and CART, in human neuronal cells [4]. This result implies that IH inhibits appetite and food intake in SAS patients by increasing mRNAs for *POMC* and *CART* in the central nervous system (CNS). However, appetite and food intake are controlled by not only the CNS but also the gastrointestinal (GI) tract, both of which work together as the gut-brain axis representing a bi-directional signaling axis. Gut peptides, which are released from enteroendocrine cells lying within the epithelium throughout the GI tract, might activate vagal and spinal afferents indirectly via activation of neurons of the enteric nervous system (ENS) and relay nutrient-derived energy signals to the brain so that appetite and food intake could be regulated appropriately through the gut-brain axis [5]. Accordingly, we hypothesized that IH could have an anorexigenic effect on the ENS, in addition to the CNS. In the present study, using enteroendocrine cell lines and an in vitro IH system, which is a controlled gas delivery system that regulates the flow of nitrogen and oxygen to generate IH, we investigated the effect of IH, a hallmark of SAS, on the gene expression(s) of major appetite-inhibiting gut peptide hormones *peptide YY* (*PYY*), *glucagon-like peptide-1* (*GLP-1*), and *neurotensin* (*NTS*) and subsequently explored their gene regulatory mechanism in human enteroendocrine cells exposed to IH.

## 2. Results

### 2.1. IH Significantly Increased the mRNA Levels of PYY, GLP-1, and NTS in Enteroendocrine Cells

**Figure 1:**
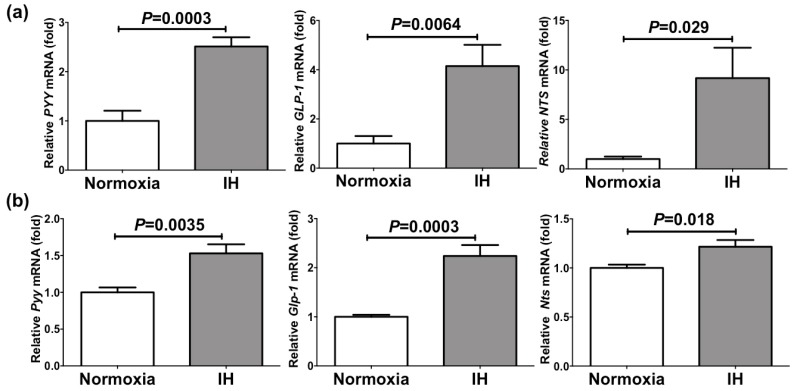
(**a**) The mRNA levels of *peptide YY* (*PYY*), *glucagon-like peptide-1* (*GLP-1*), and *neurotensin* (*NTS*) in Caco-2 cells treated with normoxia or intermittent hypoxia (IH). (**b**) The mRNA levels of *Pyy*, *Glp-1*, and *Nts* in STC-1 cells treated with normoxia or IH. The mRNA levels were measured by real-time RT-PCR and normalized by *β-actin* (for human Caco-2 cells) or *Rig/ribosomal protein S15* (*RpS15*) (for mouse STC-1 cells) as an internal standard. Data are expressed as the mean±SE of the samples (*n =* 6). Statistical analyses were performed using Student’s *t*-test. IH significantly increased the mRNA levels of *PYY*, *GLP-1*, and *NTS* in both Caco-2 and STC-1cells.

We exposed human enteroendocrine Caco-2 cells and mouse enteroendocrine STC-1 cells to IH [64 cycles of 5 min hypoxia (1% O_2_) and 10 min normoxia (21% O_2_)] or normoxia for 24 h, and measured the mRNA levels of *PYY*, *GLP-1*, and *NTS* by real-time RT-PCR. IH significantly increased the mRNA levels of *PYY* (2.51 fold, *p =* 0.0003), *GLP-1* (4.16 fold, *p =* 0.0064), and *NTS* (9.18 fold, *p =* 0.029) in Caco-2 cells and those of *Pyy* (1.53 fold, *p =* 0.0035), *Glp-1* (2.24 fold, *p =* 0.0003), and *Nts* (1.22 fold, *p =* 0.018) in STC-1 cells (Figure 1).

This result indicates that IH-stress up-regulates the mRNA levels of *PYY*, *GLP-1*, and *NTS*, which are appetite inhibitory hormones, in enteroendocrine cells, suggesting that SAS patients’ appetite could be suppressed by IH, caused by SAS, in the ENS.

### 2.2. IH Significantly Increased Secretion of PYY, GLP-1, and NTS from Caco-2 Cells

**Figure 2:**
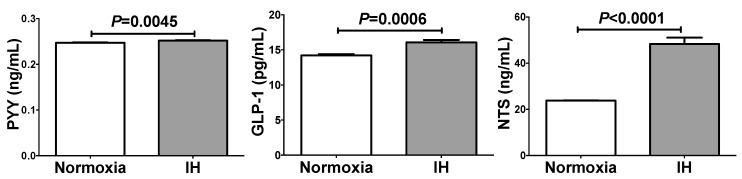
The concentrations of PYY, GLP-1, and NTS in the Caco-2 cell culture medium. IH significantly increased PYY (102 ± 0.368%), GLP-1 (113 ± 2.36%), and NTS (203 ± 11.5%) concentrations in the culture medium. The living cell numbers of normoxia treated cells and IH treated cells are similar levels because the mRNA levels of house keeping genes in IH treated cells were similar to those in normoxia treated cells (91.12 ± 4.196%, *p =* 0.1484). The measurable ranges were 0.15–10 ng/mL for PYY, 3.91–250 pg/mL for GLP-1, and 0.1–1000 ng/mL for NTS. We measured the concentrations of the peptides directly without any concentrations and dilutions. As the values were within the measurable ranges, the concentrations in the medium are comparable with those in human plasma. Data are expressed as mean±SE for each group (*n =* 6). The statistical analyses were performed using Student’s *t*-test.

We next measured the concentrations of PYY, GLP-1, and NTS in the Caco-2 cell culture medium by enzyme-linked immunosorbent assay (ELISA) and found that all of them were significantly increased by IH (Figure 2). Although the increases were statistically significant, the increases of PYY and GLP-1 were not so great compared to the differences shown in mRNA levels. As dipeptidyl peptidases were expressed in Caco-2 cells [6,7] and that PYY and GLP-1 are rapidly inactivated by dipeptidyl peptidases [8,9], PYY and GLP-1, produced and secreted from Caco-2 enterendocrine cells, could be rapidly degraded in the medium.

### 2.3. The Promoter Activities of PYY, Glucagon (GCG), and NTS Were Not Up-Regulated by IH in Caco-2 Cells

**Figure 3:**
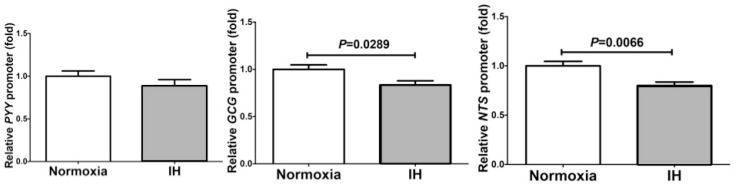
Promoter assays for *PYY*, *GCG* and *NTS*. The promoter activities of *PYY*, *GCG,* and *NTS* were not up-regulated by IH in Caco-2 cells. Caco-2 cells were transfected with constructs containing the *PYY* promoter (−2694 to +72), the *GCG* promoter (−1100 to +125), and the *NTS* promoter (−2938 to +209) upstream of a firefly luciferase reporter gene in the pGL4.17 vector. After the treatment with normoxia or IH, the luciferase activity was measured. All data are expressed as the mean±SE for each group (*n =* 6). The statistical analyses were performed using Student’s *t*-test.

To determine whether the IH-induced increases in PYY, GLP-1, and NTS in enteroendocrine cells were caused by transcriptional activation, we prepared the reporter plasmids by inserting the *PYY*, *GCG* (which encodes a preprotein, part of which is cleaved into GLP-1), and *NTS* promoter fragments upstream of a firefly luciferase reporter gene in the pGL4.17 vector, transfected them into Caco-2 cells, and measured the luciferase activity after the IH treatment. The promoter activity of *PYY* was not increased by IH, and *GCG* and *NTS* promoter activities were actually decreased by IH in Caco-2 cells (Figure 3).

### 2.4. MicroRNA-Mediated Mechanism Was Not Involved in the IH-Induced Up-Regulation of PYY, GLP-1, and NTS

**Figure 4:**
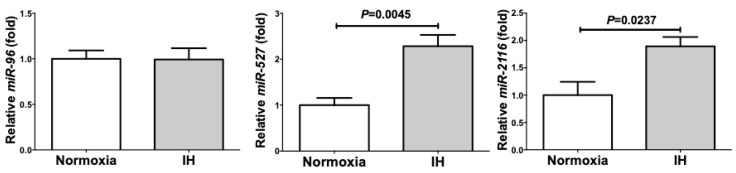
The levels of miR-96, −527, and −2116 of the IH-treated Caco-2 cells. The levels of miR-96, −527, and −2116 were measured by real-time RT-PCR using *U6* RNA as an endogenous control. Data are expressed as mean ± SE for each group (*n =* 4). The statistical analyses were performed using Student’s *t*-test.

The results of Figure 3 indicated that the IH-induced up-regulation of *PYY*, *GLP-1*, and *NTS* mRNAs was not regulated by transcriptional activation of the genes. Therefore, we first tested a hypothesis that the IH-induced up-regulation of *PYY*, *GLP-1*, and *NTS* is regulated post-transcriptionally. We searched targeted miRNA using the MicroRNA.org program (http://www.microrna.org/microrna/home.do) (23 March 2019), which revealed that *PYY*, *GLP-1,* and *NTS* mRNAs have a potential target sequence for microRNA (miR)-96, -527, and -2116, respectively. We then measured the levels of miR-96, -527, and -2116 of IH-treated cells by real-time RT-PCR and found that no miR level was decreased by IH (Figure 4).

### 2.5. Trichostatin A (TSA) Significantly Up-regulated the mRNA Levels of PYY, GLP-1, and NTS in the Normoxia Condition and 5-Azacytidine (5AZC) Significantly Decreased the mRNA Levels of PYY, GLP-1, and NTS in the IH Condition

**Figure 5:**
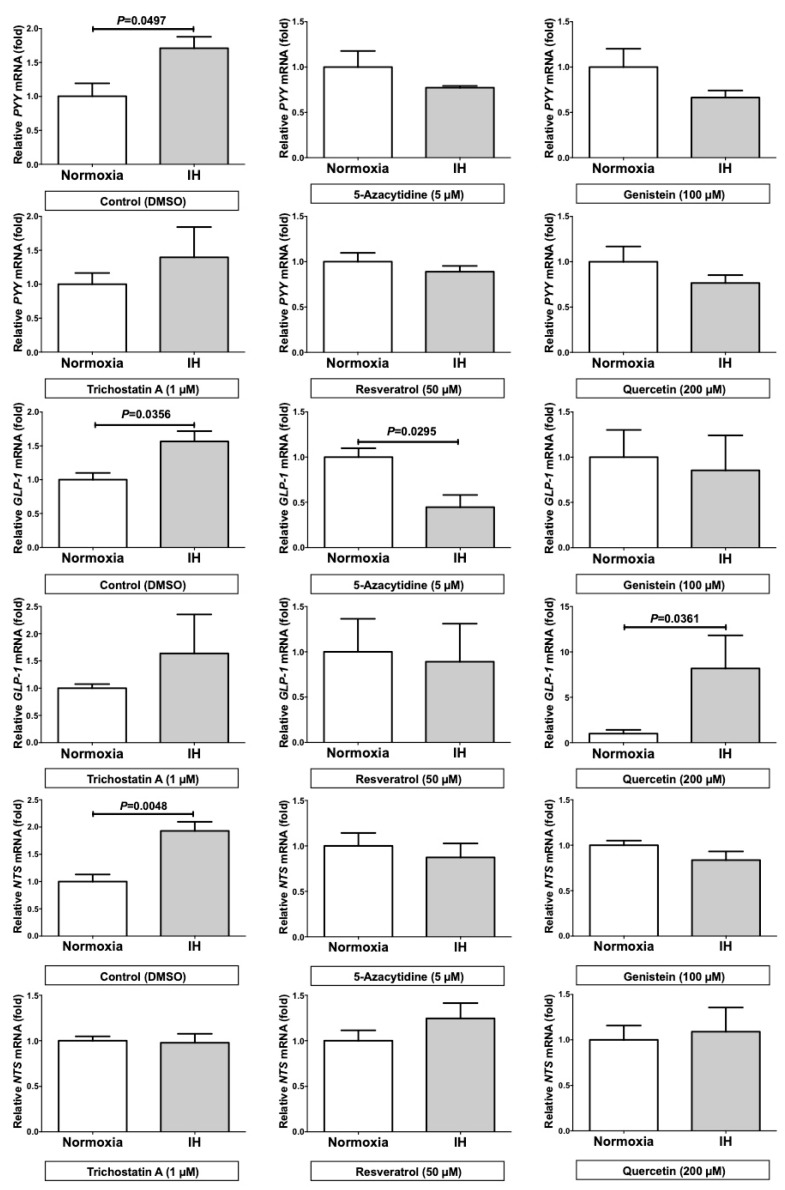
The mRNA levels of *PYY*, *GLP-1*, and *NTS* in Caco-2 cells treated with normoxia or IH under the addition of epigenetic regulators. The addition of the epigenetic regulators abolished the IH-induced up-regulation of *PYY*, *GLP-1*, and *NTS* mRNAs in Caco-2 cells. All data are expressed as the mean±SE for each group (*n =* 3–4). The statistical analyses were performed using Student’s *t*-test.

We next considered a possibility that the promoter assays did not reflect the chromatin structure of nuclear DNAs, which can alter the transcriptional efficiency, and speculated that IH might affect the chromatin structure of the genes, resulting in the up-regulation of *PYY*, *GLP-1*, and *NTS* mRNAs. To confirm whether the up-regulation of *PYY*, *GLP-1*, and *NTS* mRNAs are caused by changes in chromatin structure of the genes, we treated Caco-2 cells with 5-azacytidine (5AZC), genistein, trichostatin A (TSA), resveratrol, and quercetin, which are agents affecting the epigenetic regulation of gene expression by modifying the chromatin structure of nuclear DNAs. Six hours after the treatment, we exposed the cells to IH or normoxia for 24 h and analyzed the mRNA levels of *PYY*, *GLP-1*, and *NTS*. The addition of these drugs abolished the IH-induced up-regulation of *PYY*, *GLP-1*, and *NTS* mRNAs in Caco-2 cells (Figure 5), which implied that the IH-induced increase of *PYY*, *GLP-1*, and *NTS* mRNAs could be regulated by alterations in chromatin structures of the genes.

A comparison of the IH-group with the normoxia-group showed that the treatment with TSA significantly up-regulated the mRNA levels of *PYY* (3.578 ± 0.5476 fold), *GLP-1* (1.855 ± 0.3097 fold), and *NTS* (46.12 ± 3.310 fold) even in the normoxia condition and that the treatment with 5AZC significantly decreased the mRNA levels of *PYY* (0.749 ± 0.0590 fold), *GLP-1* (0.240 ± 0.0460 fold), and *NTS* (0.343 ± 0.0529 fold) in the IH condition.

**Figure 6:**
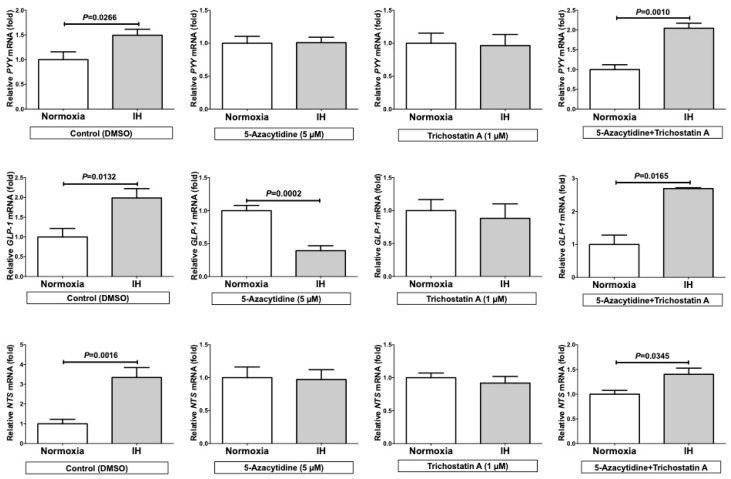
The mRNA levels of *PYY*, *GLP-1*, and *NTS* under the addition of 5-azacytidine (5AZC), trichostatin A (TSA), and 5AZC+TSA. The addition of TSA only and 5AZC only attenuated the IH-induced up-regulation of *PYY*, *GLP-1*, and *NTS*. On the other hand, combined addition of TSA+5AZC recovered the IH-induced up-regulation of *PYY*, *GLP-1*, and *NTS* mRNA levels in Caco-2 cells. All data are expressed as the mean±SE for each group (*n =* 4–8). The statistical analyses were performed using Student’s *t*-test.

Despite of the cancelation of the IH-induced up-regulation of *PYY*, *GLP-1*, and *NTS* mRNAs, by up-regulating the mRNAs in either normoxia or IH by the addition of TSA alone or down-regulating the mRNAs in IH by the addition of 5AZC alone, the combined treatment of TSA+5AZC recovered the IH-induced up-regulation of *PYY*, *GLP-1*, and *NTS* mRNAs in Caco-2 cells (Figure 6). These results indicate that the IH-induced up-regulation of *PYY*, *GLP-1*, and *NTS* could be caused by alteration in chromatin structure of the genes and that TSA has an effect similar to IH, and 5AZC have an effect opposite to IH on *PYY*, *GLP-1*, and *NTS* mRNA expressions.

## 3. Discussion

Accumulating evidence indicates a strong relationship between obesity and SAS [3]. Obesity can cause SAS for the anatomical reason that airway narrowing induced by an excess of fat tissue around the neck can lead to airway obstruction [10]. On the other hand, Li et al. described that BMI was significantly lower in SAS Far-East Asian men than in SAS white men when controlled for sex, age, and disease severity and that the mean BMI of the Far-East Asian men with SAS was below the norms for men in the United States [11]. Therefore, it remains unclear whether SAS patients’ body weight and appetite are increased because their regulation is complicated and multifactorial. With respect to the question of whether SAS patients’ appetite and food intake is increased, we previously verified the effect of IH, a hallmark of SAS, on the expression(s) of major appetite regulatory neuropeptide and receptor genes such as *POMC*, *CART*, *GAL, GALP*, *GHRL*, *QRFP*, *AGRP*, *NPY*, and *MC4R*, revealing the up-regulation of anorexigenic *POMC* and *CART* mRNAs by IH [4]. Our previous result seemed to indicate that appetite and food intake in SAS patients are suppressed in the CNS, although they are often obese. Besides the CNS, in the present study, we focused on the involvement of the ENS governing the enteroendocrine cells, where gut peptide hormones play an appetite-regulating role in the gut-brain axis. Although much attention has recently been paid to gut peptide hormones in association with metabolic disorder including diabetes and obesity, there is no report that investigated the effect of IH on the expression of gut peptide genes in enteroendocrine cells. To further investigate the anorexigenic impact of IH on appetite regulation, in the present study, we analyzed the mRNA levels of gut peptides PYY, GLP-1, and NTS, which are appetite-inhibiting hormones. PYY is released from L cells in response to intestinal nutrients and *Pyy^−/−^* mice are hyperphagic [12]. GLP-1 is implicated as a satiation signal by an increasing amount of evidence [5]. Nts is reported to be coexpressed with Pyy and Glp-1 in rodent enteroendocrine cells and to co-act with PYY and GLP-1 with respect to inhibition of gastric emptying [13]. Using human and mouse enteroendocrine cells and in vitro IH system, we here demonstrated that IH up-regulated *PYY*, *GLP-1*, and *NTS* gene expressions in enteroendocrine cells, suggesting that IH observed in SAS patients could inhibit their appetite and food intake in the ENS in addition to the CNS. IH might have an anorexigenic influence on the gut-brain axis.

On the mechanism by which IH up-regulated the mRNA levels of *PYY*, *GLP-1*, and *NTS*, the promoter assays indicated no IH-induced activation of the reporter activity for *PYY*, *GLP-1*, and *NTS* although the endogenous transcripts from chromosomal genes were increased. Therefore, we first speculated that IH up-regulated *PYY*, *GLP-1*, and *NTS* mRNAs post-transcriptionally. We actually searched targeted miRNA using the MicroRNA.org program (http://www.microrna.org/ microrna/home.do), which revealed that *PYY*, *GLP-1* and *NTS* mRNAs have potential target sequences for miR-96, miR-527, and miR-2116. There were no other miRNA candidates targeting these genes. We measured the miR-96, miR-527, and miR-2116 levels of the IH-treated cells by real-time RT-PCR. It was revealed that the level of miR-96 was not changed and that the levels of miR-527 and miR-2116 of the IH-treated cells were significantly higher than those of normoxia-treated cells (Figure 4).

**Figure 7:**
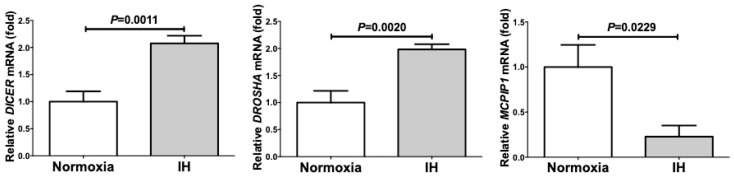
The mRNA levels of ribonuclease type III (*DROSHA*), endoribonuclease Dicer (*DICER*), and monocyte chemotactic protein-induced protein 1 (*MCPIP1*) of IH-treated Caco-2 cells by real-time RT-PCR. IH significantly increased the mRNA levels of *DROSHA* and *DICER*, which are involved in the biosynthesis of miRNAs, and significantly decreased the mRNA level of *MCPIP1*, which is involved in the degradation of miRNAs, in Caco-2 cells. All data are expressed as the mean±SE for each group (*n =* 5–6). The statistical analyses were performed using Student’s *t*-test.

We also measured the mRNA levels of ribonuclease type III (*DROSHA*), endoribonuclease Dicer (*DICER*), and monocyte chemotactic protein-induced protein 1 (*MCPIP1*) and found that IH significantly increased the mRNA levels of *DROSHA* and *DICER*, which are involved in the biosynthesis of miRNAs, and significantly decreased the mRNA level of *MCPIP1*, which is involved in the degradation of miRNAs, in Caco-2 cells (Figure 7).

These findings showed that the miRNAs, playing an important role in the degradation of mRNAs, were up-regulated by IH, indicating that the miRNA-mediated mechanism is not a reasonable explanation for the IH-induced up-regulation of *PYY*, *GLP-1*, and *NTS* mRNAs. Next, we considered that in general, promoter assays do not reflect the chromatin structure of targeted genes, which can affect the transcriptional efficiency. To confirm whether IH could cause significant chromatin remodeling, we treated cells with 5AZC, genistein, TSA, resveratrol and quercetin, which are agents affecting the epigenetic regulation of gene expression. Five-azacytidine is a DNA methyltransferase inhibitor [14]. Genistein is also a DNA methyltransferase inhibitor [15]. TSA is a histone deacetylase inhibitor [16]. Resveratrol affects epigenetic mechanisms related to DNA methyltransferase, histone deacetylase and lysine-specific demethylase-1 [17,18]. Quercetin affects epigenetic mechanisms including modulation of the DNA methylation status and histone acetylation [19]. Using these agents, we here revealed that the mRNA levels of *PYY*, *GLP-1*, and *NTS* were significantly up-regulated by the TSA-addition compared with no addition in normoxia-condition and were significantly decreased by the 5AZC-addition in IH-condition. Furthermore, the combined treatment of TSA+5AZC recovered the IH-induced up-regulation of *PYY*, *GLP-1*, and *NTS* mRNAs, suggesting that the combination of TSA and 5AZC canceled each effect by changing chromatin structure. From these results, we concluded that the IH-induced up-regulation of *PYY*, *GLP-1*, and *NTS* mRNAs in enteroendocrine cells could result from alterations in chromatin structure of the genes. Furthermore, TSA could have an effect similar to IH and 5AZC could have an opposite effect on *PYY*, *GLP-1*, and *NTS* mRNA expressions. It is possible that the mRNA levels of *PYY*, *GLP-1*, and *NTS* are up-regulated by IH via alterations in their chromatin structure in enteroendocrine cells.

## 4. Materials and Methods

### 4.1. Cell Culture

Human Caco-2 enteroendocrine cells were obtained from Institute of Development, Aging, and Cancer, Tohoku University (Sendai, Japan) and mouse STC-1 enteroendocrine cells from American Type Culture Collection (ATCC^®^; Manassas, VA, USA). They were maintained in DMEM medium (Wako Pure Chemical Industries, Ltd., Osaka, Japan) with 10% fetal calf serum (FCS), 100 units/mL penicillin G (Wako), and 100 µg/mL streptomycin (Wako). The cells were cultured at 37 °C under 5% CO_2_.

We exposed the Caco-2 and STC-1 cells to normoxia (21% O_2_, 5% CO_2_, and balance N_2_) or intermittent hypoxia (IH: 64 cycles of 5 min sustained hypoxia [1% O_2_, 5% CO_2_, and balance N_2_] and 10 min normoxia), mimicking the SAS environment in 24-well plate (1 × 10^5^ cells/mL, 0.5 mL/dish) for 24 h using a custom-designed, computer-controlled incubation chamber attached to an external O_2_–CO_2_–N_2_ computer-driven controller (O_2_ programmable control, 9200EX, Wakenyaku Co., Ltd., Kyoto, Japan) as described [2,20]. This condition is almost similar to that described in patients with a severe degree of SAS: Patients with severe SAS are repeatedly exposed to severe hypoxemia followed by mild hypoxemia or normoxic condition, that is, IH. As previously reported, the magnitude of IH expressed by peripheral oxygen saturation fluctuated between 75–98% and 50–80% in SAS patients [2]: These values were nearly equal to the medium condition in the present study [2,21].

### 4.2. Real-Time Reverse Transcription-Polymerase Chain Reaction (RT-PCR)

After the normoxia or IH treatment, total RNA was isolated from Caco-2 and STC-1 cells with an RNeasy Protect Cell Mini Kit (Qiagen, Hilden, Germany), as described previously [4,20,21,22]. The isolated RNA was reversetranscribed to the cDNA using High Capacity cDNA Reverse Transcription Kit (Applied Biosystems, Foster City, CA, USA) for real-time PCR, as described previously [4,20,23,24]. The mRNA levels of *PYY*, *GLP-1*, and *NTS* were measured by real-time RT-PCR. All the primers used for real-time RT-PCR were synthesized by Nihon Gene Research Laboratories, Inc. (NGRL; Sendai, Japan) and described in Table 1.

Real-time PCR was performed by the KAPA SYBR^®^ Fast qPCR Master Mix (Kapa Biosystems, Boston, MA, USA) and the Thermal Cycler Dice Real Time System (Takara, Kusatsu, Japan), as described previously [4,20,22,25,26]. PCR was performed with an initial step of 3 min at 95 °C followed by 45 cycles of 3 s at 95 °C and 20 s at 66 °C for h*PYY,* m*Pyy,* h*GLP-1*, and m*Glp-1*, and was conducted with an initial step of 3 min at 95 °C followed by 45 cycles of 3 s at 95 °C and 20 s at 60 °C for h*NTS*, m*Nts*, *β-actin*, and *Rig/RpS15*. Target cDNAs were cloned into pBluescript SK(-) plasmid (Stratagene, La Jolla, CA, USA), and sequential 10-fold dilutions from 10^2^~10^7^ copies/µL were prepared. The serial dilutions were run to verify the specificity and to test the sensitivity of the SYBR Green-based real-time RT-PCR. The respective mRNA levels were normalized by those of the *β-actin* mRNA for human, by those of *Rig/RpS15* mRNA for mouse mRNAs, and the miR levels were normalized by *U6* RNA level as an internal standard [4,20,22,25,26,27,28,29].

### 4.3. Measurement of PYY, GLP-1, and NTS in Culture Medium by Enzyme-Linked Immunosorbent Assay (ELISA)

After Caco-2 cells were exposed either IH or normoxia for 24 h, culture medium was collected and the concentrations of PYY, GLP-1, and NTS were measured by using a Human PYY ELISA kit (RayBio^®^, Nocross, GA, USA), a Human GLP-1 ELISA kit (Enzo Life Sci., Farmingdale, NY, USA), and a Human NTS ELISA kit (RayBio^®^) according to the instructions of suppliers, respectively.

### 4.4. Construction of Reporter Plasmids and Luciferase Reporter Assay

Reporter plasmids were constructed by inserting deleted fragments of the *PYY* promoter (−2694 to +72), the *GCG* promoter (−1100 to +125), and the *NTS* promoter (−2938 to +209) upstream of a firefly luciferase reporter gene in the pGL4.17[*luc2*/Neo] vector (Promega, Madison, WI, USA). Caco-2 cells were seeded in a 24-well plate (1 × 10^5^ cells/mL, 0.5 mL/dish) and were transfected with reporter plasmids by Lipofectamine^®^3000 (Life Technologies, Carlsbad, CA, USA), as described previously [4,23,24,29]. After the treatment with IH or normoxia for 24 h, the cells were harvested and cell extracts were prepared in Extraction Buffer (0.1 M potassium phosphate, pH 7.8/0.2% Triton X-100; Life Technologies). To monitor transfection efficiency, the pCMV-SPORT-βgal plasmid (Life Technologies) was co-transfected in all experiments at a 1:10 dilution. Luciferase activity was measured by using the PicaGene Luciferase assay system (Toyo-ink, Tokyo, Japan) and normalized by the β-galactosidase activity, as previously described [4,20,28,29,30].

### 4.5. Treatment of Caco-2 cells with 5AZC, Genistein, TSA, Resveratrol and Quercetin

Epigenetic regulators, 5AZC (Tokyo Chemical Industry Co., Ltd., Tokyo, Japan), genistein (Wako), TSA (Tokyo Chemical Industry Co., Ltd.), resveratrol (Wako), and quercetin (Wako), were dissolved by DMSO. Cells were plated in 24-well plates and were incubated for 24 h. After the incubation, 5 µM of 5AZC, 100 µM of genistein, 1 µM of TSA, 50 µM of resveratrol, and 200 µM of quercetin were added to each well, while DMSO was added as a control at 1% final concentration. Six hours after the addition of these agents, cells were exposed to IH or normoxia for 24 h and were harvested. Total RNA was extracted and real-time RT-PCR was conducted as described.

### 4.6. Data Analysis

All the values were expressed as the mean ± SE. The data obtained were checked by Shapiro-Wilk normality test and found that all the *p* values were larger than 0.05, and then they were analyzed by Student’s *t*-test using the GraphPad Prism6 software (GraphPad Software, La Jolla, CA, USA). A *p* value of <0.05 was considered statistically significant.

## 5. Conclusions

IH significantly up-regulated *PYY*, *GLP-1*, and *NTS* gene expressions in enteroendocrine cells. TSA has an effect similar to IH and 5AZC has an effect opposite to IH on the regulation of *PYY*, *GLP-1*, and *NTS* mRNAs in Caco-2 cells, suggesting that IH-stimulation may have some epigenetic regulatory effects on the gene expression of *PYY*, *GLP-1*, and *NTS*. It is possible that the IH-induced up-regulation of PYY, GLP-1, and NTS might be regulated by alteration in chromatin structure of the genes.

## Figures and Tables

**Table 1:** PCR primers for real-time RT-PCR.

Target mRNA	Primer Sequence (Accession Number: Position)
h*PYY*	5′-TATGGTGTTCGTGCGCAGGC-3′ (NM_004160: 541-560)
	5′-TCACCACAGGTCTGGGCCCTC-3′ (NM_004160: 815-835)
m*Pyy*	5′-GCAGCGGTATGGAAAAAGAGA-3′ (NM_145435: 219-239)
	5′-TCCAAACCTTCTGGCCTGAA- 3′ (NM_145435: 304-323)
h*GLP-1*	5′-TGTCAGCGTAATATCTGTGAGGC-3′ (NM_002054: 86-108)
	5′-AGCAGGTGAAGAGAGAGCAAGC-3′ (NM_002054: 201-222)
m*Glp-1*	5′-GCACATTCACCAGCGACTACA-3′ (NM_008100: 267-287)
	5′-GCAATGTTGTTCCGGTTCCT-3′ (NM_008100: 347-366)
h*NTS*	5′-CAGCAGGGCTTTTCAACACTGGG-3′ (NM_006183: 445-467)
	5′-TCTCTTTTGAGTATGTAGGGTCTTCTGGG-3′ (NM_006183: 578-606)
m*Nts*	5′-CTGGTGTGCCTGACTCTCCT-3′ (NM_024435: 117-136)
	5′-TCACATCTTCTTCTGAATCTGAGC-3′ (NM_024435: 160-183)
DROSHA	5′-GGCCCGAGAGCCTTTTATAG-3′ (NM_013235: 167-186)
	5′-TGCACACGTCTAACTCTTCCAC-3′ (NM_013235: 263-284)
DICER	5′-GAGCTGTCCTATCAGATCAGGG-3′ (NM_177438: 476-497)
	5′-ACTTGTTGAGCAACCTGGTTT-3′ (NM_177438: 547-567)
MCPIP1	5′-TGCCTATCACAGACCAGCAC-3′ (NM_025079: 722-741)
	5′-CTCACCTTCGCGAAGTAGCTC-3′ (NM_025079: 913-933)
miR-96	5′-TGGCCGATTTTGGCACTAGCAC-3′ (NR_029512: 1-22)
	5′-TTCCCATATTGGCACTGCACATGA-3′ (NR_029512: 54-77)
miR-527	5′-CTCAAGCTGTGACTGCAAA-3′ (NR_030219: 2-20)
	5′-TCTCAAACCGTAATTCACCAAA-3′ (NR_030219: 64-85)
miR-2116	5′-CTAGGGGTTCTTAGCATAGG-3′ (NR_031750: 9-28)
	5′-CTAGTCTGGGAGTTCTTGG-3′ (NR_031750: 60-78)
*β-actin*	5′-GCGAGAAGATGACCCAGA-3′ (NM_001101: 420-437)
	5′-CAGAGGCGTACAGGGATA-3′ (NM_001101: 492-509)
m*Rig/RpS15*	5′-ACGGCAAGACCTTCAACCAG-3′ (NM_009091: 323-342)
	5′-ATGGAGAACTCGCCCAGGTAG-3′ (NM_009091: 372-392)
U6	5′-CTCGCTTCGGCAGCACA-3′ (NR_004394)
	5′-AACGCTTCACGAATTTGCGT-3′ (NR_004394)

## Abbreviations

5AZC	5-Azacytidine
AGRP	Agouti-related peptide
CART	Cocaine- and amphetamine-regulated transcript
CNS	Central nervous system
DICER	Endoribonuclease Dicer
DROSHA	Ribonuclease type III
ENS	Enteric nervous system
GAL	Galanin
GALP	Galanin-like peptide
GHRL	Ghrelin
GCG	Glucagon
GI	Gastrointestinal
GLP-1	Glucagon-like peptide-1
IH	Intermittent hypoxia
MC4R	Melanocortin 4 receptor
MCPIP1	Monocyte chemotactic protein-induced protein 1
NPY	Neuropeptide Y
NTS	Neurotensin
POMC	Proopiomelanocortin
PYY	Peptide YY
QRFP	Pyroglutamylated RFamide peptide
RT-PCR	Reverse transcription-polymerase chain reaction
SAS	Sleep apnea syndrome
TSA	Trichostatin A
